# Enhancement of a prime editing system via optimal recruitment of the pioneer transcription factor P65

**DOI:** 10.1038/s41467-023-35919-0

**Published:** 2023-01-17

**Authors:** Ronghao Chen, Yu Cao, Yajing Liu, Dongdong Zhao, Ju Li, Zhihui Cheng, Changhao Bi, Xueli Zhang

**Affiliations:** 1 College of Life Science, Nankai University, Tianjin, China; 2 Tianjin Institute of Industrial Biotechnology, Chinese Academy of Sciences, Tianjin, China; 3 National Technology Innovation Center of Synthetic Biology, Tianjin, China; 4 College of Life Science, Tianjin Normal University, Tianjin, China

**Keywords:** Biotechnology, CRISPR-Cas9 genome editing, Transcription factors

## Abstract

Prime editing is a versatile gene editing tool that enables precise sequence changes of all types in the genome, but its application is rather limited by the editing efficiency. Here, we first apply the Suntag system to recruit the transcription factor P65 and enhance the desired editing outcomes in the prime editing system. Next, MS2 hairpins are used to recruit MS2-fused P65 and confirmed that the recruitment of the P65 protein could effectively improve the prime editing efficiency in both the PE3 and PE5 systems. Moreover, this suggests the increased editing efficiency is most likely associated with the induction of chromatin accessibility change by P65. In conclusion, we apply different systems to recruit P65 and enhance the prime editing efficiency of various PE systems. Furthermore, our work provides a variety of methods to work as protein scaffolds for screening target factors and thus supports further optimization of prime editing systems.

## Abstract (web summary)

Prime editing represents a great advance to the genome editing field but is currently limited by the editing efficiency. Here the authors look to improve the efficiency by recruiting target proteins and show that the transcription factor P65 could enhance the desired editing outcomes at different gene loci.

## Introduction

Since a large number of human diseases are associated with genetic variants, the ability to correct genomic variants has been a long-term pursuit of researchers. Despite the rapid development of genome editing technologies in recent years, altering DNA efficiently, precisely, and freely still remains a great challenge. Based on CRISPR gene editing technology, base editing and prime editing have been developed^1^. As the next generation of precise gene editing tools, these strategies can produce point mutations without requiring additional donor DNA templates and double-strand DNA breaks (DSBs)^2^. Base editing can achieve only a few base transition and transversion types but not all types, and incurs excess byproducts when multiple target nucleotides appear in the editing window. Compared to base editing, prime editing can achieve free transformation of bases, including four types of base transitions and eight base transversion types, and can carry out the insertion and deletion of small fragments in the genome^1^. Moreover, despite the versatile editing ability, the application of prime editing is still limited by its relatively low editing efficiency.

The core prime editing (PE2) system mainly includes an editing protein and a pegRNA^2^. The editing protein was constructed by fusing nCas9(H840A) with reverse transcriptase (RT), and an additional primer-binding site (PBS) and reverse transcription template were added to the 3′ end of the regular guide RNA to obtain pegRNA. In the current model, the pegRNA forms a complex with the editing protein and guides the nCas9 enzyme to cut the targeted DNA strand containing the protospacer adjacent motif (PAM) sequence. Thereafter, the released 3′ single-stranded DNA at the nick binds to the PBS sequence designed in pegRNA, which works as a primer for the reverse transcription reaction. Then, the 3′ single-stranded DNA is extended by using the RT template region of the pegRNA. Consequently, the 3′ flap containing the desired edits competes with the original unedited 5′ flap to hybridize with the non-PAM containing the DNA strand via a flap equilibrium mechanism. Usually, the excision of the 5′ flap by an intracellular 5′ exonuclease induces the ligation of the edited sequence into the genome. To optimize the prime editing system, David Liu’s group constructed a PE3 system by nicking the nonediting strand using a simple sgRNA, which was thought to direct DNA repair to use the edited strand as a template^2^. In 2021, they used CRISPRi to systematically analyze the endogenous factors affecting the effect of prime editing and found that inhibiting the DNA mismatch repair (MMR) pathway can enhance the efficiency and accuracy of prime editing by coexpressing the dominant negative MMR protein MLH1dn; thus, they developed PE4 systems^3^. By integrating the PE3 and PE4 systems, the PE5 (PE3 + MLH1dn) system was constructed^3^. Moreover, by incorporating a structured RNA motif into the 3′ end of pegRNA to enhance its stability, they also developed epegRNA, which greatly improved the editing efficiency^4^.

The current research to advance prime editing focuses on the structural optimization of pegRNA and transient expression of related proteins^5–8^. Theoretically, direct protein fusion can make the protein work in the editing region precisely and efficiently^9,10^. Thus, protein fusion is a well-established method of modulating genome editing outcomes. For example, the fusion of uracil glycosylase inhibitor (UGI) with an editing protein can improve editing efficiency and optimize cytosine base editing (CBE), and fusion of two copies of the UGI domain can further increase the activity of CBE^11^. In addition to direct protein fusion, some other strategies have been applied for recruiting the target protein to a genetic locus. As reported, some researchers have recruited multiple target proteins via the Suntag system, in which Cas9-fused GCN4 peptides can recruit multiple copies of single-chain variable fragment (scFv)-fused target proteins^12,13^. Moreover, a single guide RNA (sgRNA) bearing an MS2 hairpin binding site has been previously used to recruit MS2-fused effector proteins. In the MS2 system, the sgRNA contains two MS2 hairpins in its stem loop, which recruit two target effector proteins fused to the MS2 proteins (four in total)^14^.

In this study, we initially found that the direct fusion of proteins with the prime editing protein significantly reduced the protein’s editing efficiency. Next, we optimized the Suntag system in the prime editing system to recruit the target protein and found that fusion of two copies of GCN4 peptides had the least impact on the prime editing system. Furthermore, the transcription factor P65 was screened through the Suntag system and used to enhance editing efficiency at different genomic loci with the PE system. In addition, MS2 hairpins were used to recruit MS2-fused P65 and confirmed that the recruitment of the P65 protein can effectively improve the prime editing efficiency in the PE3 and PE5 systems. Thus, we applied the Suntag and MS2 systems to recruit P65 in prime editing systems and enhanced prime editing outcomes.

## Results

### Direct fusion of cell factors to the prime editor decreased the editing efficiency

Theoretically, Fen1, a 5′ flap endonuclease, has been proposed to play roles in 5′ flap excision during prime editing^2^. Consistent with this hypothesis, knockdown of Fen1 reduces the frequency of the intended editing, suggesting that the activity of Fen1 promotes the desired editing outcomes^3^. Motivated by these results, we sought to construct prime editors through direct protein fusion of Fen1, which would facilitate the function of the endonuclease in the target region. We first fused Fen1 to the N-terminus and C-terminus of the PE2 editor; the resulting constructs were designated Fen1-PE2 and PE2-Fen1, respectively (Supplementary Fig. 1A). We then assessed the editing outcomes across three genomic loci in HEK293T cells. Compared to the original PE2, we observed that the N-terminus-fused editor PE2-Fen1 showed a significant reduction in editing efficiency at all three genomic loci. However, the Fen1 fusion to the C-terminus of PE led to a more dramatic decrease in editing efficiency than the fusion to the N-terminus (Supplementary Fig. 1B). Next, we fused T5 exonuclease, which can degrade single-stranded DNA in the 5′→3′ direction, to the N-terminus of PE2 and named the resulting construct T5exo-PE2. Similar to Fen1-PE2, T5exo-PE2 also showed a greatly reduced editing efficiency compared with that of PE2 at all three sites (Supplementary Fig. 2B).

To verify whether the editing efficiency reduction was caused by the protein function or spatial structure, we fused different pioneer transcription factor genes into the PE2-expressing vectors. Pioneer transcription factors can bind to nucleosomes, loosen the dense chromatin structure and allow Cas9 to bind to the corresponding site, thus promoting editing efficiency. Here, the pioneer transcription factors FOXA1 (52 kD) and VP64 (5.5 kDa) were fused to the editors for the construction of FOXA1-PE2 and VP64-PE2, respectively. We compared the editing efficiencies of PE2, VP64-PE2, and FOXA1-PE2 (Supplementary Fig. 2A) and found that the editors with the smaller pioneer transcription factor VP64 showed a weaker tendency toward decreased efficiency (Supplementary Fig. 2B). We consequently reasoned that the size of the fused protein has a negative impact on the editing outcomes. Taken together, these data suggested that the direct fusion of various proteins reduced the editing frequency of prime editing and that employing the protein interactions to recruit the target proteins might be a better alternative.

### Adapting the Suntag system for screening of determinants of editing outcomes

As mentioned previously, in the Suntag system, GCN4 is a tandem repeatable polypeptide containing 19 amino acids that can be specifically recognized by single-chain variable fragment antibodies (scFvs)^13^. One study has shown that the combination of the GCN4 peptide and scFv system with a base editing system can broaden the editing window and improve editing accuracy. We hypothesized that this system would allow us to fuse a repeating GCN4 peptide array with a PE editor, with the aim of recruiting multiple copies of scFv-fusion proteins.

**Fig. 1:**
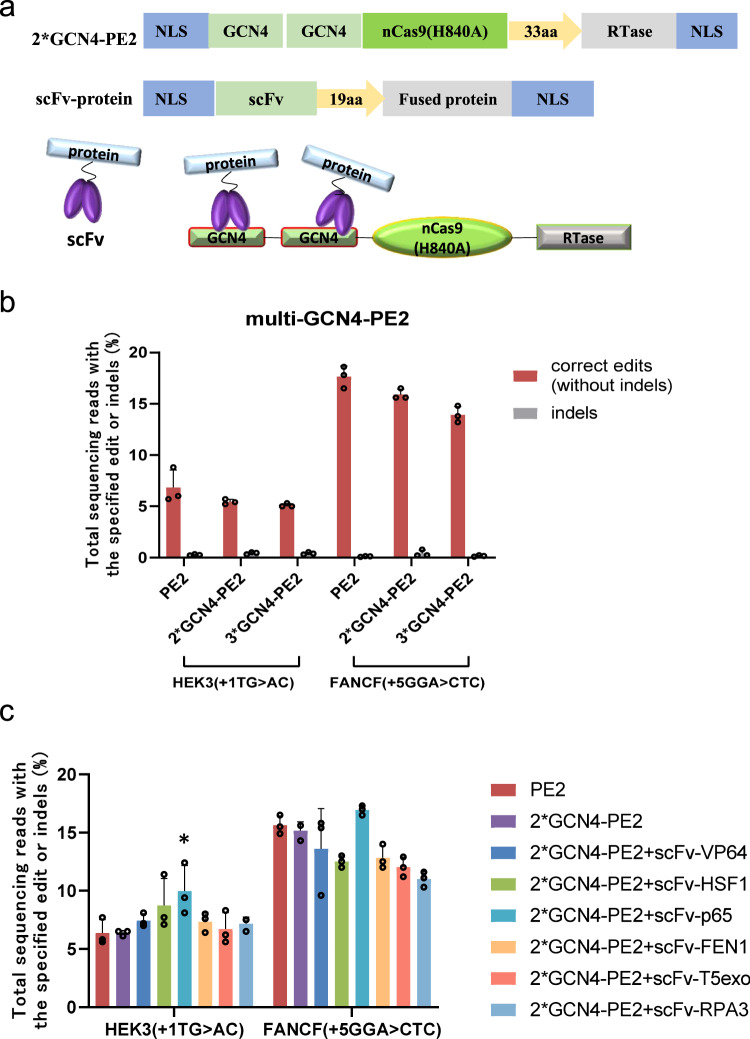
Adapting the Suntag system for screening of determinants of editing outcomes. **a** Construction design of Suntag-based prime editing systems. **b** Editing efficiency of 2*GCN4-PE2 and 3*GCN4-PE2. Two or three copies of the GCN4 peptides were fused to the N-terminus of the nCas9 enzyme, and the resulting constructs were named 2*GCN4-PE2 and 3*GCN4-PE2, respectively. At the two genomic loci of HEK3 and FANCF, the editing efficiencies induced by prime editors with the increased copy number of GCN4 were determined. The gray bar indicates the indel frequency coupled with the editing efficiency indicated by the left closest bar. Bars represent the mean of *n* = 3 independent replicates. The error bars in all figures represent the SDs. **c** Editing efficiency of 2*GCN4-PE2 with various cell factors. In the screening experiment, plasmids expressing scFv-linked target proteins were constructed and delivered into HEK293T cells with the 2*GCN4-PE2 expression vector. The editing efficiencies at the two genomic loci, HEK3 and FANCF, were measured. Bars represent the mean of *n* = 3 independent replicates. The error bars in all figures represent the SDs. The *P* values were calculated by a two-tailed *t* test. *P < 0.05. Source data are provided as a Source Data file.

Considering the negative impact of protein fusion on the prime editors, we optimized the copy number of GCN4 for the maximal recruitment of targeting proteins with minimal negative impact of fusion. Due to the relatively small molecular size of the GCN4 peptide, two and three copies of the GCN4 peptide were fused to the N-terminus of the nCas9 enzyme, and the resulting constructs were named 2*GCN4-PE2 and 3*GCN4-PE2, respectively (Fig. 1a). At the two genomic loci of HEK3 and FANCF, the editing efficiencies decreased, corresponding with the increase in the copy number of GCN4 (Fig. 1b), as expected. This correlation between editing efficiencies and GCN4 copy numbers again indicated the negative impact of the fusion of larger peptides to the prime editor.

As 2*GCN4-PE2 showed similar or slightly decreased editing efficiency, 2*GCN4-PE2 was chosen as the protein scaffold to screen the target factors, which might promote editing activity. According to the prime editing model, Fen1 endonuclease, T5 exonuclease, and replication protein A (RPA), which can specifically bind to the 3′ end of single-stranded DNA, are proposed to facilitate 5′ flap excision and 3′ flap protection^3,15,16^. The pioneer transcription factors HSF1, VP64, and P65 can bind to nucleosomes and loosen the dense chromatin structure^17–19^. In the effector screening experiments, we constructed plasmids expressing scFv-linked target proteins and then delivered them into HEK293T cells with the 2*GCN4-PE2 expression vector with PEI reagent. Among the six cellular factors, we found that the prime editor with the transcription factor P65 was highly effective at HEK3 site (Fig. 1c).

### P65 improves prime editing at different genomic loci in the PE system

**Fig. 2:**
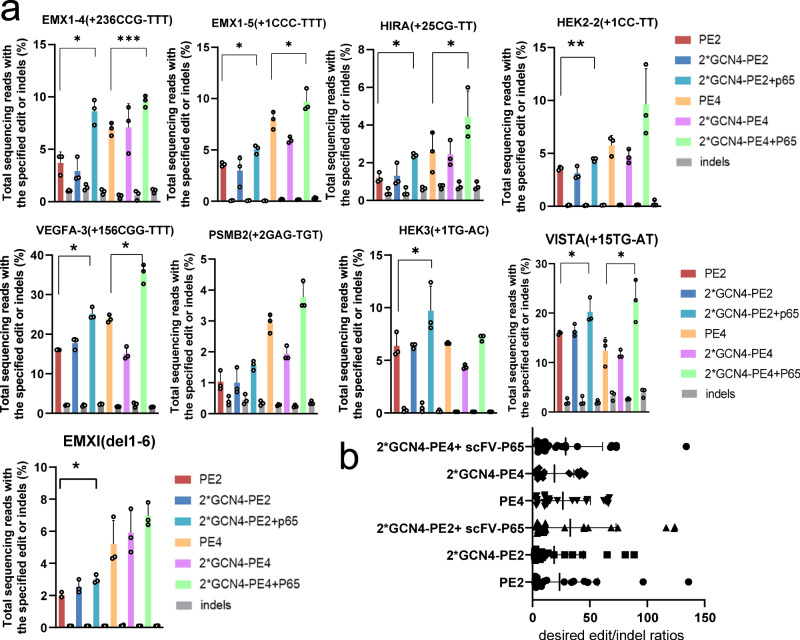
P65 improved PE2 prime editing efficiency at various genomic loci. **a** PCR amplicons from the target regions were analyzed by targeted deep sequencing. The reads harboring only correct edits were counted to evaluate the editing efficiency, and the reads harboring any undesired insertion or deletion were counted to evaluate the indel frequency. The gray bar indicates the indel frequency coupled with the editing efficiency indicated by the left closest bar. Statistical analysis of the normalized increases in intended editing efficiencies. Bars represent the mean of *n* = 3 independent replicates. The error bars in all figures represent the SDs. The *P* values were calculated by a two-tailed *t* test. ***P < 0.005; **P < 0.01; *P < 0.05. **b** Summary of PE2 and PE4 editing enhancement with the recruitment of P65. Bars represent the mean of *n* = 3 independent replicates. The error bars in all figures represent the SDs. Source data are provided as a Source Data file.

To validate the above results, we further assessed the effect of P65 on PE system with base-substitution edits and deletion edits at more genomic loci, including EMX1, PSMB2, HEK2, HIRA, VEGFA, and VISTA. We first transfected HEK293T cells with vectors encoding 2*GCN4-PE2, P65-scFv, and pegRNAs and measured prime editing results by deep sequencing. Compared with the original PE2 system, the PE2 Suntag system that recruited the P65 protein through protein interaction was observed to have increased editing efficiency at eight out of the nine tested loci. The PE2 Suntag system increased the editing efficiency on average by 1.65-fold, at EMX1 site 4 by 2.3-fold, at EMX1 site 5 by 1.39-fold, at HIRA site by 2-fold, at HEK2 site 2 by 1.24-fold, at VEGFA site 3 by 1.92-fold, at HEK3 site by 1.54-fold, at the VISTA site by 1.27-fold, and at the EMX1 site by 1.5-fold, respectively (Fig. 2a). Furthermore, the PE4 Suntag system that recruited the P65 protein enhanced average editing efficiency over PE4 by 1.55-fold at five out of the nine tested loci (Fig. 2a). Importantly, this system increased editing efficiency and did not increase indel byproducts. The recruitment of P65 increased edit/indel ratios by 1.4-fold in PE2 system and 1.16-fold in PE4 system, respectively (Fig. 2b). In addition, the PE5 Suntag system also showed an increased editing efficiency at two tested loci (Supplementary Fig. 3). The editing results of these genomic loci showed that the recruitment of the P65 protein by Suntag could effectively improve the prime editing outcomes without increasing indels.

To evaluate whether this recruitment of P65 could influence off-target editing activity, we treated HEK293T cells with original or modified PE2 and PE4 systems and measured the resulting genomic changes at the four most common Cas9 off-target sites (Supplementary Table 2) for each targeted locus^2^. As shown in Supplementary Fig. 4, the average frequency of off-target prime editing remained at a very low level with or without the recruitment of P65. These results indicated that the recruitment of P65 via Suntag system did not increase off-target prime editing.

Moreover, we directly fused the pioneer transcription factor P65 to the editor, designated P65-PE2. Recent studies fused Rad51 into the middle Cas9 nickase and RT domains and generally increased the PE editing efficiencies^20^. Inspired by this work, we constructed PE2-mid P65 (Supplementary Fig. 5A). Compared with the original PE2, the editing efficiencies of P65-PE2 and PE2-mid P65 were decreased at all three tested loci, including EMX1 site 4, FANCF, and EMX1 site 5, which was consistent with the previous editing results (Supplementary Fig. 5B). Taken together, these results further supported the idea that direct protein fusion of P65 to prime editors impeded prime editing, and that recruitment of P65 by the Suntag system could promote prime editing outcomes.

### Recruitment of P65 through the MS2 system

**Fig. 3:**
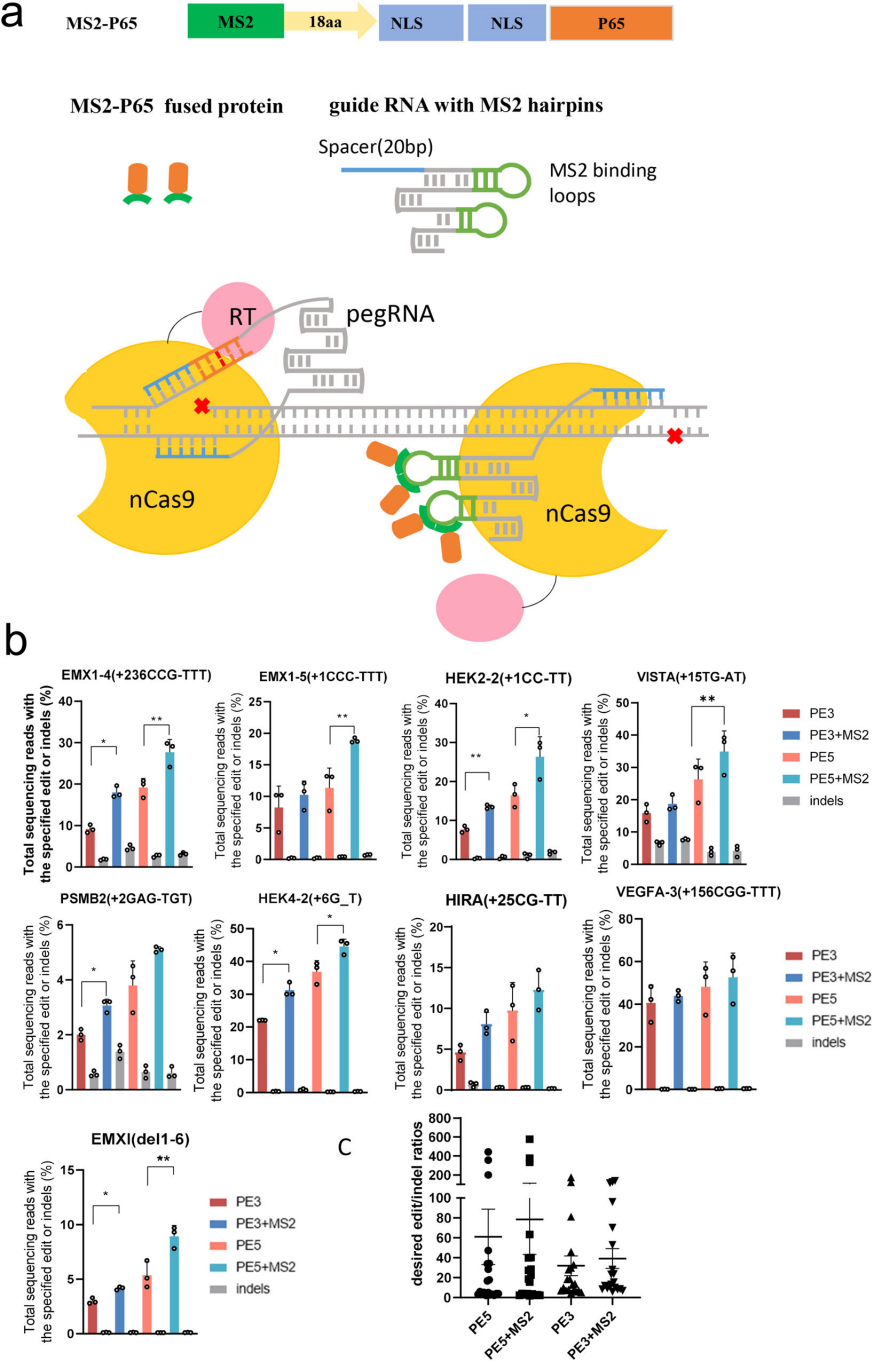
Editing efficiency of the PE3 and PE5 systems upon recruitment of P65 through the MS2 system. **a** Diagrams of the MS2-P65 fusion protein constructs and an overview of the MS2-P65-recruiting PE3 or PE5 (PE3 + MLH1dn) system. **b** Prime editing of genomic DNA in HEK293T cells by the MS2-P65-recruiting PE3 and PE5 systems. The editing efficiencies reflect the sequencing reads that contained the intended edits and did not contain indels among all treated cells, with no sorting. The gray bar indicates the indel frequency coupled with the editing efficiency indicated by the left closest bar. Bars represent the mean of *n* = 3 independent replicates. The error bars in all figures represent the SDs. The *P* values were calculated by a two-tailed *t* test. **P < 0.01; *P < 0.05. **c** Summary of PE3 and PE5 editing enhancement with the recruitment of p65. Bars represent the mean of *n* = 3 independent replicates. The error bars in all figures represent the SDs. Source data are provided as a Source Data file.

To apply different approaches in recruiting the P65 protein to a genetic locus, we initially used prime editors combined with pegRNA harboring an MS2 hairpin binding site that has been previously used to recruit MS2-fused effector proteins. In this system, we generated a pegRNA containing MS2 hairpins in its stem loop and an MS2 protein fused to P65. However, the prime editing system with the reconstructed pegRNAs that contained MS2 hairpins decreased the editing efficiency (Supplementary Fig. 6). This might have been due to the change in pegRNA structure. Since the PE3 system employs an additional gRNA to cleave the non-PAM strand, we modified the second guide RNA of the PE3 system by inserting two MS2 hairpin copies into the conventional sgRNAs, which should have also facilitated the targeting of the fusion proteins of P65 and MS2 to the targeted gene loci and was predicted to increase the editing efficiencies (Fig. 3a).

To test the above hypothesis, prime editing experiments were carried out with the original PE3 system and MS2-P65 recruiting PE3 system at different genomic loci. The editing results in HEK293T cells were obtained by deep sequencing. The MS2-P65 recruiting PE3 system outperformed the PE3 system in inducing intended editing outcomes and increasing the efficiencies at five out of nine loci, from 9.1 to 18.0% at EMX1 site 4, 7.7 to 13.5% at HEK2 site 2, 2.0 to 3.1% at PSMB2, 22.0 to 31.2% at HEK4 site 2, and 3.0 to 4.2% at EMX1 site 1 (Fig. 3b).

To further assess this improved prime editing system, we replaced the second guide RNA with an MS2-modified guide RNA in the PE5 (PE3 + MLH1dn) system to obtain the MS2-modified PE5 system to recruit MS2-P65 fusion proteins. Next, we used the PE5 system and MS2-P65-recruited PE5 system to edit eight genomic sites. Relative to the PE5 system with normal guide RNA, the MS2-P65-recruited PE5 system enhanced editing efficiency by an average of 1.37-fold in HEK293T cells (Fig. 3b). The MS2-P65-recruited PE3 and PE5 systems increased edit/indel ratios over original PE3 and PE5 systems by 1.3-fold and 1.23-fold, respectively (Fig. 3c). Moreover, the off-target editing efficiency remained at low level with or without P65, which suggested that the recruitment of P65 via MS2 system did not affect the off-target editing efficiency (Supplementary Fig. 7).

Collectively, these results demonstrate that the recruitment of P65 by the MS2 system enabled increased editing efficiency and outcomes with various PE systems.

### Recruitment of p65 promotes the chromatin accessibility

**Fig. 4:**
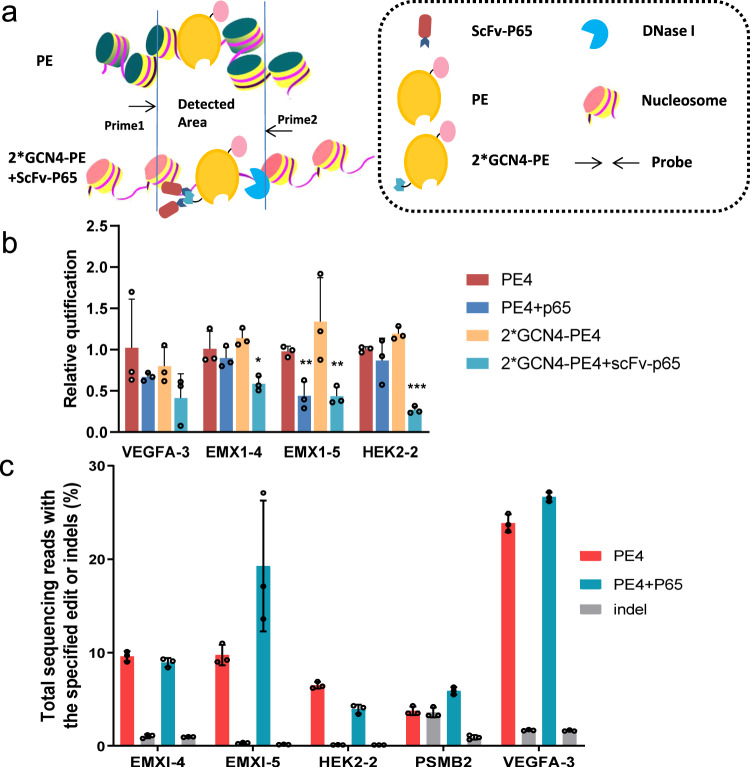
Recruitment of P65 promotes chromatin accessibility at target genome loci. **a** Schematic of functional mechanism of P65 recruited prime editors and DNase I digestion assays. **b** Low-input DNase I digestion assays were performed for the detection of chromatin accessibility at four genomic sites in HEK293T cells. Bars represent the mean of *n* = 3 independent replicates. The error bars in all figures represent the SDs. The *P* values were calculated by a two-tailed *t* test. *P < 0.05. **c** PE editing efficiencies in the presence of overexpressed P65 were tested. Bars represent the mean of *n* = 3 independent replicates. The error bars in all figures represent the SDs. Source data are provided as a Source Data file.

The transcriptional activation was reported to modulate chromatin accessibility and induce an open chromatin environment^21–23^. Current evidence points towards chromatin states playing a significant role in Cas9 binding and functioning^23^. Furthermore, Gal4-p65 fusions were reported to open closed chromatin, generate a CRISPR-accessible state and enhance CRISPR/Cas9 editing efficiency^24^. To investigate the possible molecular mechanisms underlying the increased editing efficiency in P65-associated PE systems, we tested the alteration of chromatin accessibility at different chromatin regions. The DNase I assay was performed to detect the alteration of chromatin states at four genomic sites (Fig. 4a). The chromosomal DNA samples were harvested and subjected to DNase I digestion, followed by amplification of the analyzed region with specific PCR primers, as shown in Fig. 4a. Typically, the open region is more exposed to DNase I, receives more digestion, and has fewer intact DNA cassettes remaining, thus resulting in a lower concentration of PCR product. Our results showed that the recruitment of P65 induced increased chromatin accessibility at the targeted loci of PE, compared to the control with no transcription factor p65 (Fig. 4b). While compared with the recruited P65 proteins, the free overexpression of P65 did not affect the chromatin accessibility at three out of the four tested loci. Additionally, we examined the PE editing efficiencies in the presence of the overexpression of the P65 at different genomic loci. The data showed that overexpression of p65 protein had only a slight effect on editing efficiency at indicated tested loci (Fig. 4c).

Taken together, these results indicated that the recruitment of P65 could promote chromatin accessibility effectively, and their function in prime editing was most likely associated with the induction of chromatin accessibility.

## Discussion

The current research for the improvement of prime editing efficiency mainly focused on the structural optimization of pegRNA and transient expression of MMR inhibitory proteins^5–8^. Since peptide and protein fusion is a well-established method allowing the protein to work in the editing region precisely and efficiently, and modulate genome editing outcomes^9,10^, we initially constructed prime editors directly fused with different factors. However, almost all constructed prime editors were found to have reduced editing efficiency.

Recent studies showed that the direct fusion of Rad51 DNA-binding domain^20^ or various peptides^25,26^ to PE2 generally increases the PE editing efficiencies. For the fusion of the Rad51 DNA-binding domain, the authors inserted Rad51 DBD into the region between nCas9 and RT domains, N- or C-terminus of PE2, generating variants named PE2-mid_Rad51, PE2-N_Rad51, and PE2-C_Rad5, respectively. PE2-N_Rad51 and PE2-C_Rad51 showed lower activities in many loci sites, which was similar to our results. The PE2-mid_Rad51 showed an obvious increase in editing efficiencies; however, this type of fusion pattern may not be versatile for other proteins, such as the PE2-mid RPA70 in the same paper^20^ and PE-mid_P65 (Supplementary Fig. 5). For the fusion of different peptides to PE2^25,26^, similar to GCN4, these peptides show a slight effect on PE system. Taken together, these studies appear contradictory but are consistent with our results.

As known, editing efficiency can be affected by many other factors, such as time points and puromycin addition^20,23,27^. Our previous study has also shown that the editing outcome differs significantly at different time points (e.g., 24, 48, 72, 96, 120, 144 h) in one base editing process^27^. Generally, adding puromycin into the culture medium was for removing cells not transfected with the editing plasmids. The addition of antibiotics is a common protocol in genomic editing research, which is mainly to ensure the tested cells contain the editing plasmids and increase the uniformity of the editing efficiency data^20^. To minimize other errors, we performed all prime editing experiments strictly according to the protocol as described in “Methods”.

To optimize the outcomes of protein fusion, we applied various strategies in recruiting the protein to the target genetic locus instead of a direct protein fusion. First, we applied the Suntag system in the PE2 system to recruit the target protein and found that the fusion of two copies of GCN4 peptides showed a slight impact on the prime editing system. Furthermore, the pioneer transcription factor P65 was selected by the Suntag system and used to enhance the editing efficiency of various PE systems. Moreover, MS2 hairpins were used to recruit MS2-fused P65, and the recruitment of P65 protein effectively improved the prime editing efficiency in the PE3 and PE5 systems. With the DNase I assay, it was also proved the increased editing efficiency was most likely associated with the induction of chromatin accessibility by P65. In conclusion, we applied the Suntag and MS2 systems to recruit the transcription factor P65 in various prime editing systems and enhanced the prime editing outcomes. In addition, our work provides a variety of methods for the recruitment of proteins to work as protein scaffolds for the screening of target factors and thus supports further optimization of prime editing systems.

## Methods

### Plasmid construction

All plasmids were assembled by the Golden Gate method. Guide RNA expression plasmids were assembled with the Golden Gate method with the N20 sequence (Supplementary Table 1) embedded in the primers. The PCR primers (Tsingke, China) for pegRNA were designed with the desired sequences (PBS and RT template sequence) embedded in the primers. All the DNA templates were PCR amplified with PrimeSTAR HS DNA polymerase (Takara, Japan). The PCR products were gel purified by Sanprep Gel Extraction kit (Sangon, China), digested with DpnI restriction enzyme (NEB, USA), and assembled via the Golden Gate assembly method.

### Cell culture and transfection

HEK293T cells (from ATCC) were cultured in Dulbecco’s minimal essential medium (DMEM, Gibco) supplemented with 10% (vol/vol) fetal bovine serum (FBS) and 1 × penicillin streptomycin (Corning). The cells were incubated and cultured at 37 °C with 5% CO_2_. Before transfection, the cells were seeded in 24-well plates (Corning), incubated for approximately 24 h and then transfected with PEI after reaching approximately 40% confluence. A total of 600 ng of Cas9 plasmid and 300 ng of pegRNA-expressing plasmid or 100 ng of additional scFV-expressing plasmid was transfected into cells in 150 μl of DMEM containing 3 μl of PEI. Twenty-four hours after transfection, 4 μg/ml puromycin (Merck) was added to the medium. the cells were incubated for another 4 days and then collected for high-throughput sequencing.

### High-throughput sequencing

Total genomic DNA was extracted using QuickExtract DNA extraction solution (Epicenter, USA) supplemented with proteinase K (Roche) following the manufacturer’s instructions with slight modifications. Cells were washed with PBS and lysed in 30 μl of extraction solution supplemented with 0.3 μl of proteinase K. The samples were incubated at 55 °C for 10 min and inactivated at 80 °C for 3 min. Targeted regions (180–250 bp) of interest were amplified by PCR with indicated primers (Supplementary Table 2) and used for high-throughput DNA sequencing. Libraries with different barcodes were analyzed by Illumina high-throughput sequencing (GENEWIZ, China). The data were split according to their barcodes, and the examined target sites were selected by Rgenome. The base-substitution ratios were calculated by dividing the base-substitution reads by the total reads.

### Detection of chromatin accessibility

Low-input DNase I digestion assays were performed for the detection of chromatin accessibility^21^. The cells were seeded and incubated for approximately 24 h and then transfected with PEI after reaching approximately 40% confluence. Then, 6 × 10^5^ transfected HEK293T cells were collected and resuspended in 50 μL lysis buffer and incubated on ice for 5 min. After that, DNase I (Sigma, USA) was added to the samples and further incubated at 37 °C for 5 min. Finally, the reaction was terminated with 50 μL stop buffer at 55 °C for 1 h. The genomic DNA was extracted via the phenol–chloroform method and ethanol precipitated. The purified DNA samples were analyzed by real-time qPCR (SYBR GREEN, TOYOBO, Japan) using the LightCycler 96 System with three replicates. The relative quantification of intact DNA at each indicated locus was calculated using the genomic site of gapdh as the internal reference. The relative quantified values are used to compare the difference and change between the test group and control group. The primers used are listed in Supplementary Table 3.

### Statistics and reproducibility

Statistical analyses were performed using Graphpad Prism 8. The data are shown as means ± SD from three independent experiments. The bars represent the mean values, and the error bars represent the SDs. The *P* values were calculated by a two-tailed *t* test. ***P < 0.005; **P < 0.01; *P < 0.05. *P* values less than 0.05 were considered statistically significant.

### Reporting summary

Further information on research design is available in the Nature Portfolio Reporting Summary linked to this article.

## Supplementary information

Supplementary Information

Reporting Summary

## Data Availability

There is no restriction on the data associated with this study. High-throughput sequencing data have been deposited in the NCBI database: PRJNA854910 and PRJNA892719. Source data are provided with this paper.

## Source data

Source data
