# Supplementary material for: Enhancement of a prime editing system via optimal recruitment of the pioneer transcription factor P65
Source: Nat Commun. 2023 Jan 17;14:257. doi: 10.1038/s41467-023-35919-0 (PMC9845348; doi:10.1038/s41467-023-35919-0)
Supplement: Supplementary file 1 — Supplementary Information [file 41467_2023_35919_MOESM1_ESM.pdf]

Supplementary information for

Enhancement of a prime editing system via optimal recruitment of the pioneer transcription factor P65

Chen, *et. al*

1. **Supplementary Fig. 1. Direct fusion of FEN1 to the prime editor decreased intended editing outcomes in HEK293T cells.**
2. **Supplementary Fig. 2. The size of the fused protein has a differential negative impact on the editing outcomes.**
3. **Supplementary Fig. 3. Editing efficiency of the PE5 systems upon recruitment of P65 through the Suntag system.**
4. **Supplementary Fig. 4. Off-target prime editing from PE2 and PE4 in HEK293T cells.**
5. **Supplementary Fig. 5. Direct fusion of P65 to the prime editor decreased intended editing outcomes in HEK293T cells.**
6. **Supplementary Fig. 6. The prime editing system with the reconstructed pegRNAs (containing MS2 hairpins) decreased the editing efficiency rapidly in HEK293T cells.**
7. **Supplementary Fig. 7. Off-target prime editing from PE3 and PE5 in HEK293T cells.**
8. **Supplementary Table 1. pegRNA and gRNA sequence.**
9. **Supplementary Table 2. deep sequencing primers.**

10. Supplementary Table 3. qPCR primers.

Supplemental figures

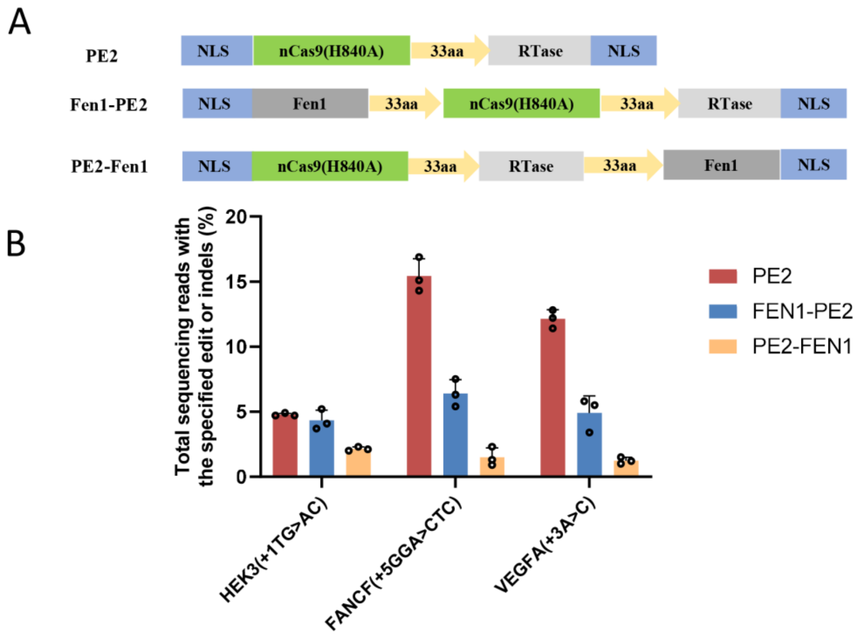

Supplementary Fig. 1. Direct fusion of FEN1 to the prime editor decreased intended editing outcomes in HEK293T cells. (A) Schematic representation of prime editors fused with FEN1 in the N-terminus or C-terminus, designated Fen1-PE2 and PE2-Fen1, respectively. (B) The editing outcomes across 3 genomic loci in HEK293T cells were assessed. Bars represent the mean of n=3 independent replicates. The error bars in all figures represent the SDs. Source data are provided as a Source Data file.

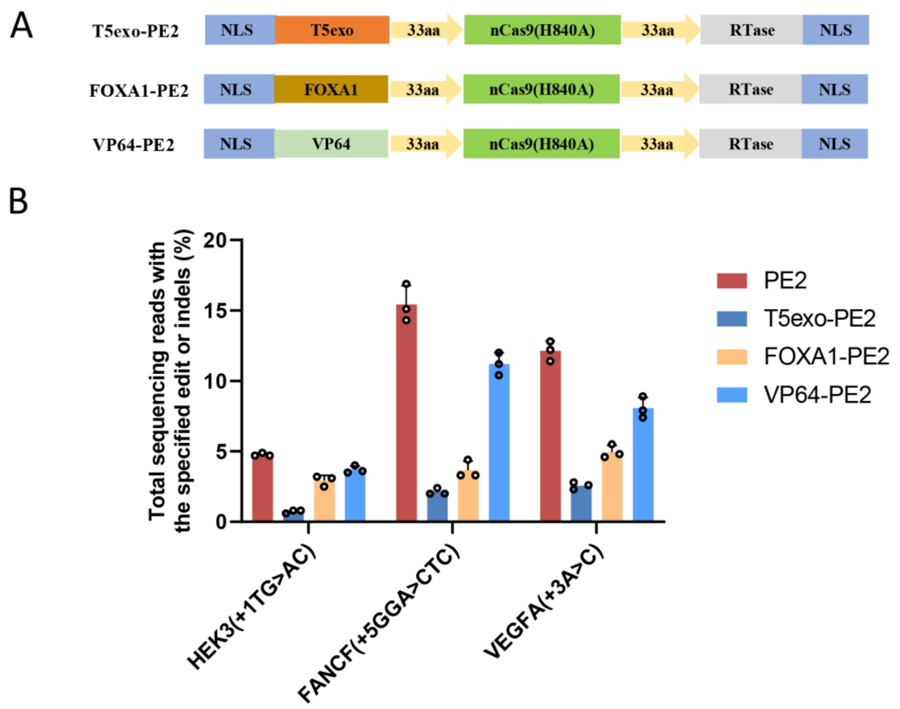

Supplementary Fig. 2. The size of the fused protein has a differential negative impact on the editing outcomes. (A) Schematic representation of various effectors fused with prime editors in the N-terminus. (B) The editing outcomes across 3 genomic loci in HEK293T cells were assessed. Bars represent the mean of n=3 independent replicates. The error bars in all figures represent the SDs. Source data are provided as a Source Data file.

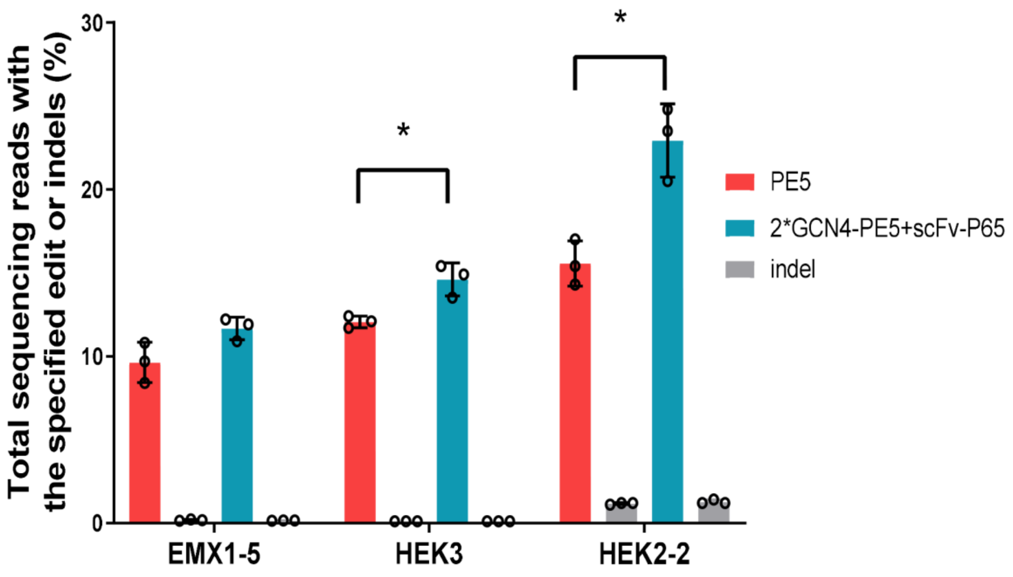

Supplementary Fig. 3. Editing efficiency of the PE5 systems upon recruitment of P65 through the Suntag system. The editing outcomes across 3 genomic loci in HEK293T cells were assessed. Bars represent the mean of n=3 independent replicates. The error bars in all figures represent the SDs from three independent biological replicates. The P values were calculated by two-tailed t test. *, P<0.05. Source data are provided as a Source Data file.

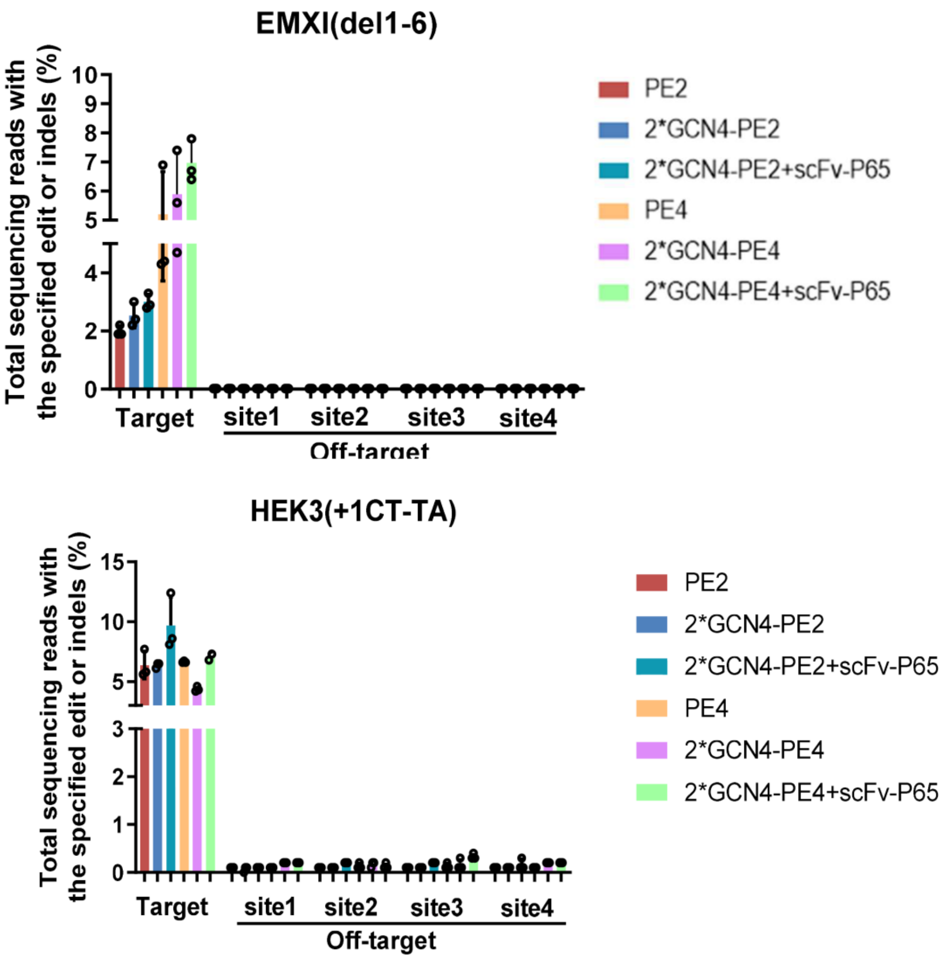

Supplementary Fig. 4. Off-target prime editing from PE2 and PE4 in HEK293T cells. Bars represent the mean of n=3 independent replicates. The error bars in all figures represent the SDs. Source data are provided as a Source Data file.

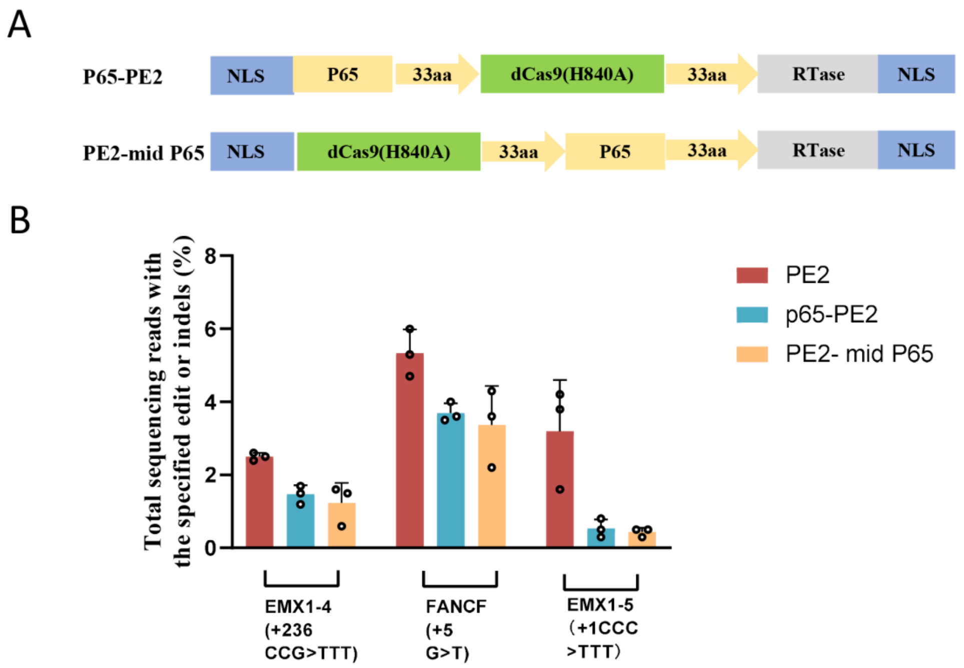

Supplementary Fig. 5. Direct fusion of P65 to the prime editor decreased intended editing outcomes in HEK293T cells. (A) The pioneer transcription factor P65 was directly fused to the N-terminus or middle site of editing protein, named as P65-PE2 and PE2-mid P65, respectively. (B) The editing outcomes across different genomic loci in HEK293T cells were assessed. Bars represent the mean of n=3 independent replicates. The error bars in all figures represent the SDs. Source data are provided as a Source Data file.

A

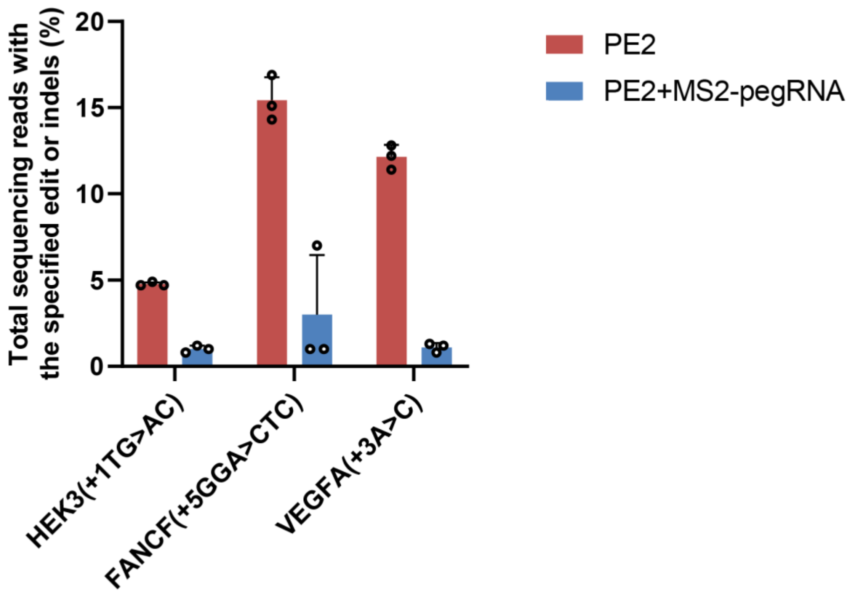

Supplementary Fig. 6. The prime editing system with the reconstructed pegRNAs (containing MS2 hairpins) decreased the editing efficiency rapidly in HEK293T cells. In this system, pegRNA harboring MS2 hairpin binding sites was used to edit the indicated genomic loci. Bars represent the mean of n=3 independent replicates. The error bars in all figures represent the SDs. Source data are provided as a Source Data file.

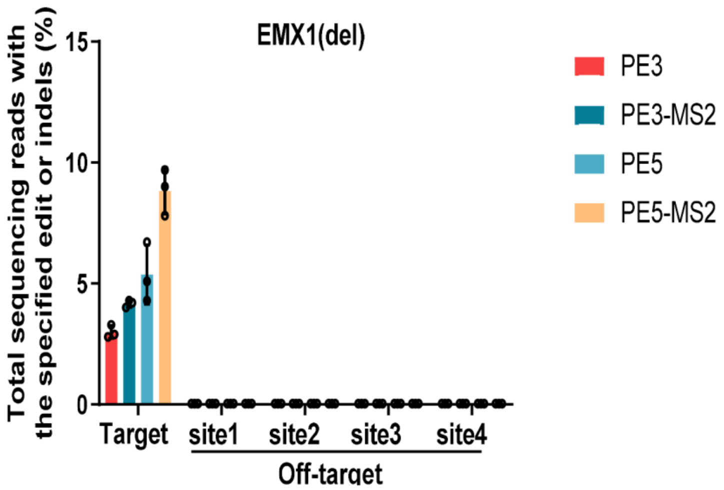

Supplementary Fig. 7. Off-target prime editing from PE3 and PE5 in HEK293T cells. Bars represent the mean of n=3 independent replicates. The error bars in all figures represent the SDs. Source data are provided as a Source Data file.

Supplementary Table 1. pegRNA and gRNA sequence.

pegRNA	spacer sequence	3’extension sequence（5’→3’）	PBS length(nt)	RT template length(nt)
EMX1_del+1-6	GAGTCCGAGCAGAAGAAGAA	TGATGTGATGGGAGTTCTTCTGCTCGG	13	14
EMX1_site4_+236CCG>TTT	GTATTCATTTCCCTAGCTCC	TAGCCGTCACTAAAGCTAGGGAAATGAATA	16	14
EMX1_site5_+123CCC>TTT	GCTAACTTACTGTGTAACCC	TGACTTGCCCAAAATTACACAGTAAGTTA	15	14
FANCF_+567GGA>CTC	GGAATCCCTTCTGCAGCACC	GGAAAAGCGAGAGAGGTGCTGCAGAAGGGAT	14	17
HEK3_+12TG>AC	GGCCCAGACTGAGCACGTGA	TCTGCCATGTCGTGCTCAGTCTG	13	10
HEK4_OT2_+6G>T	GTACACTTGTGCAACCTCAC	TAGAGTTACAGTGAGGTTGCACAAGTG	14	13
HIRA_site1_+25CG>TT	GTATTCTAGAATGCAGGGCA	CCTGTTTCCACTACCCTGCATTCTAGAAT	15	14
PSMB2_+234GAG>TGT	GTAAACAAAGCATAGACTGA	TTGTACCCACAAGTCTATGCTTTGTTT	15	12
VEGFA_site3_+156CGG>TTT	TCCCTCTTTAGCCAGAGCCG	TGCACAAACCGACTCTGGCTAAAGA	13	12
VISTA_+15TG>AT	GAACACAAAGCATAGACTGC	GGCCCGCCACGCTGTCTATGCTTTGTGT	15	13
HEK2_OT2_+12CC>TT	GAATACTTCTTCAGAGTCCC	GAGTATCCTGAAACTCTGAAGAAGTAT	15	12

PE3 nicking gRNA	spacer sequence
EMX1_gRNA_+53	GACATCGATGTCCTCCCCAT
EMX1_site6_gRNA_+66	CCACAGCTGTGACCTCAAAG
HEK2_OT2_gRNA_+26	CTTGATGGCTAGATAACTTT
HEK4_OT2_gRNA_+40	GTTTCCCCATCCATAAAGTG
HIRA_site1_gRNA_+43	GTGAAGTGCCCTTCCTCCTT
PSMB2_gRNA_+31	CATAGCTGTTGCATGAGGAA
VEGFA_site3_gRNA_+44	TTCACCCAGCTTCCCTGTGG
VISTA_gRNA_+21	GCACTTGTTTGCAGCTATTC

Supplementary Table 2. Deep sequencing primers.

editing sites and Barcode	sequence(5'-3')
EMX1-A1	CCATCAATGCCccagaaccggaggacaaagtacaaac
EMX1-A2	CGATGGCGATAccagaaccggaggacaaagtacaaac
EMX1-A3	ACACAAGCACCccagaaccggaggacaaagtacaaac
EMX1-A4	CCGTTTCGACGccagaaccggaggacaaagtacaaac
EMX1-A5	GGAAGTAGACCccagaaccggaggacaaagtacaaac
EMX1-A6	GCATTATCTATccagaaccggaggacaaagtacaaac
EMX1-A7	CCTGGTCATCGccagaaccggaggacaaagtacaaac
EMX1-A8	TCAGTCTGTGCccagaaccggaggacaaagtacaaac
EMX1-reverse	caagcagcactctgccctcgtgggtttg
FANCF-B1	CAGAGGCCGATagagtcgccgtctccaaggtgaaag
FANCF-B2	CACTTTGCTCCagagtcgccgtctccaaggtgaaag
FANCF-B3	CATGCTTTCGAagagtcgccgtctccaaggtgaaag
FANCF-B4	CTGCTCTTTTTagagtcgccgtctccaaggtgaaag
FANCF-B5	CTCAGACGGTGagagtcgccgtctccaaggtgaaag
FANCF-B6	CTTTGCGACCAagagtcgccgtctccaaggtgaaag
FANCF-B7	CTAGCTGGTGTagagtcgccgtctccaaggtgaaag
FANCF-B8	AGGATCGGAGTagagtcgccgtctccaaggtgaaag
FANCF-reverse	tgcgaatggggccatgccgaccaaag
HEK3-C1	TGATGGGAGAGagagaagcctggagacagggatcccag
HEK3-C2	ACCAGATTGACagagaagcctggagacagggatcccag
HEK3-C3	ACGACTGAATCagagaagcctggagacagggatcccag
HEK3-C4	AGCTTGTTGCTagagaagcctggagacagggatcccag
HEK3-C5	AGTACAGGTTAagagaagcctggagacagggatcccag
HEK3-C6	ACTCCGTCGGGagagaagcctggagacagggatcccag
HEK3-C7	AGAACGGCTCTagagaagcctggagacagggatcccag
HEK3-C8	AGTTAGTTATCagagaagcctggagacagggatcccag
HEK3-reverse	ctgtctaggaaaagctgtcctgcgacg
EMX1-site4-A1	CCATCAATGCCccctcattcctgggaatctcttg
EMX1-site4-A2	CGATGGCGATAccctcattcctgggaatctcttg
EMX1-site4-reverse	cagtttcggactaggacagttagc
EMX1-site5-A1	CCATCAATGCCgacagagcttcccaggtgggcaaac
EMX1-site5-A2	CGATGGCGATAgacagagcttcccaggtgggcaaac
EMX1-site5-reverse	gctactaatctgacatactaccttaaagggaagg
EMX1-site6-A1	CCATCAATGCCggcaaagggtacagaaacgtggcctgtg
EMX1-site6-A2	CGATGGCGATAggcaaagggtacagaaacgtggcctgtg
EMX1-site6-C1	TGATGGGAGAGggcaaagggtacagaaacgtggcctgtg
EMX1-site6-C2	ACCAGATTGACggcaaagggtacagaaacgtggcctgtg
EMX1-site6-reverse	aggcctcctggagccatcttctcaagc
HEK2-site2-A1	CCATCAATGCCtctgggagtgcagtaatg
HEK2-site2-A2	CGATGGCGATAtctgggagtgcagtaatg
HEK2-site2-C1	TGATGGGAGAGtctgggagtgcagtaatg
HEK2-site2-C2	ACCAGATTGACtctgggagtgcagtaatg

HEK2-site2-reverse	cctcccttagagttaagc
PSMB2-A1	CCATCAATGCCagctgttgcatgaggaaagg
PSMB2-A2	CGATGGCGATAagctgttgcatgaggaaagg
PSMB2-A3	ACACAAGCACCagctgttgcatgaggaaagg
PSMB2-A4	CCGTTTCGACGagctgttgcatgaggaaagg
PSMB2-reverse	gccgtgggagacaattcatatc
VISTA-A1	CCATCAATGCCGGCTGAGCTAACTGTGACAGCATGTG
VISTA-A2	CGATGGCGATAGGCTGAGCTAACTGTGACAGCATGTG
VISTA-C1	TGATGGGAGAGGGCTGAGCTAACTGTGACAGCATGTG
VISTA-C2	ACCAGATTGACGGCTGAGCTAACTGTGACAGCATGTG
VISTA-reverse	CCCAAGTGAGAAGCCAGTGGAATAC
HEK4-OT2-A1	CCATCAATGCCgtgtgatcaactgcaataag
HEK4-OT2-A2	CGATGGCGATAgtgtgatcaactgcaataag
HEK4-OT2-A3	ACACAAGCACCgtgtgatcaactgcaataag
HEK4-OT2-A4	CCGTTTCGACGgtgtgatcaactgcaataag
HEK4-OT2-reverse	tggagcctcatacactataac
HIRA-1-A1	CCATCAATGCCGGGGACCAGAGGTAAATAAGGC
HIRA-1-A2	CGATGGCGATAGGGGACCAGAGGTAAATAAGGC
HIRA-1-C1	TGATGGGAGAGGGGGACCAGAGGTAAATAAGGC
HIRA-1-C2	ACCAGATTGACGGGGACCAGAGGTAAATAAGGC
HIRA-1-reverse	CTAGTCACGTCAAGGTTCAGTCC
**Off-target primers**	
HEK3 off-target site 1 fwd -A1	CCATCAATGCCTCCCCTGTTGACCTGGAGAA
HEK3 off-target site 1 fwd -A2	CGATGGCGATATCCCCTGTTGACCTGGAGAA
HEK3 off-target site 1 rev	CACTGTACTTGCCCTGACCA
HEK3 off-target site 2 fwd A1	CCATCAATGCCTTGGTGTTGACAGGGAGCAA
HEK3 off-target site 2 fwd A2	CGATGGCGATATTGGTGTTGACAGGGAGCAA
HEK3 off-target site 2 rev	CTGAGATGTGGGCAGAAGGG
HEK3 off-target site 3 fwd A1	CCATCAATGCCTGAGAGGGAACAGAAGGGCT
HEK3 off-target site 3 fwd A2	CGATGGCGATATGAGAGGGAACAGAAGGGCT
HEK3 off-target site 3 rev	GTCCAAAGGCCCAAGAACCT
HEK3 off-target site 4 fwd A1	CCATCAATGCCTCCTAGCACTTTGGAAGGTCG
HEK3 off-target site 4 fwd A2	CGATGGCGATATCCTAGCACTTTGGAAGGTCG
HEK3 off-target site 4 rev	GCTCATCTTAATCTGCTCAGCC
EMX1 off-target site 1 fwd A1	CCATCAATGCCGTGGGGAGATTTGCATCTGTGGAGG
EMX1 off-target site 1 fwd A2	CGATGGCGATAGTGGGGAGATTTGCATCTGTGGAGG
EMX1 off-target site 1 rev	GCTTTTATACCATCTTGGGGTTACAG
EMX1 off-target site 2 fwd A1	CCATCAATGCCCAATGTGCTTCAACCCATCACGGC
EMX1 off-target site 2 fwd A2	CGATGGCGATACAATGTGCTTCAACCCATCACGGC
EMX1 off-target site 2 rev	CCATGAATTTGTGATGGATGCAGTCTG
EMX1 off-target site 3 fwd A1	CCATCAATGCCGAGAAGGAGGTGCAGGAGCTAGAC
EMX1 off-target site 3 fwd A2	CGATGGCGATAGAGAAGGAGGTGCAGGAGCTAGAC
EMX1 off-target site 3 rev	CATCCCGACCTTCATCCCTCCTGG
EMX1 off-target site 4 fwd A1	CCATCAATGCCGTAGTTCTGACATTCCTCCTGAGGG

EMX1 off-target site 4 fwd A2	CGATGGCGATAGTAGTTCTGACATTCCTCCTGAGGG
EMX1 off-target site 4 rev	TCAAACAAGGTGCAGATACAGCA
FANCF off-target site 1 fwdA1	CCATCAATGCCGCGGGCAGTGGCGTCTTAGTCG
FANCF off-target site 1 fwdA2	CGATGGCGATAGCGGGCAGTGGCGTCTTAGTCG
FANCF off-target site 1 rev	CCCTGGGTTTGGTTGGCTGCTC
FANCF off-target site 2 fwd A1	CCATCAATGCCCTCCTTGCCGCCCAGCCGGTC
FANCF off-target site 2 fwd A2	CGATGGCGATACTCCTTGCCGCCCAGCCGGTC
FANCF off-target site 2 rev	CACTGGGGAAGAGGCGAGGACAC
FANCF off-target site 3 fwd A1	CCATCAATGCCCCAGTGTTTCCCATCCCCAACAC
FANCF off-target site 3 fwd A2	CGATGGCGATACCAGTGTTTCCCATCCCCAACAC
FANCF off-target site 3 rev	GAATGGATCCCCCCCTAGAGCTC

Supplementary Table 3. qPCR primers.

Real-time qPCR sequence	sequence(5'-3')
VEGFA-site3 fwd	GACCACATTGTCAGAGGGACACACTG
VEGFA-site3 rev	CCCAGCTTCCCTGTGGTG
EMX1-site4 fwd	ccctcattcctgggaatctcttg
EMX1-site4 rev	cagtttcggactaggacagttagc
EMX1-site5 fwd	gacagagcttcccaggtgggcaaac
EMX1-site5 rev	taatctgacatactaccttaaagggaagg
HEK2-site2 fwd	tctgggagtgcagtaatg
HEK2-site2 rev	cctcccttagagttaagc
gapdh fwd	AGAAGGCTGGGGCTCATTTG
gapdh rev	AGGGGCCATCCACAGTCTTC
