# Supplementary material for: Enhancement of a prime editing system via optimal recruitment of the pioneer transcription factor P65
Source: Nat Commun. 2023 Jan 17;14:257. doi: 10.1038/s41467-023-35919-0 (PMC9845348; doi:10.1038/s41467-023-35919-0)
Supplement: Supplementary file 2 — Reporting Summary [file 41467_2023_35919_MOESM2_ESM.pdf]

nature portfolio

Corresponding author(s): ZHihui Cheng, Changhao Bi, Xueli Zhang

Last updated by author(s): Dec 24, 2022

Reporting Summary

Nature Portfolio wishes to improve the reproducibility of the work that we publish. This form provides structure for consistency and transparency in reporting. For further information on Nature Portfolio policies, see our Editorial Policies and the Editorial Policy Checklist.

Statistics

For all statistical analyses, confirm that the following items are present in the figure legend, table legend, main text, or Methods section.

n/a	Confirmed	
☐	☒	The exact sample size (n) for each experimental group/condition, given as a discrete number and unit of measurement
☐	☒	A statement on whether measurements were taken from distinct samples or whether the same sample was measured repeatedly
☐	☒	The statistical test(s) used AND whether they are one- or two-sided<br>*Only common tests should be described solely by name; describe more complex techniques in the Methods section.*
☒	☐	A description of all covariates tested
☒	☐	A description of any assumptions or corrections, such as tests of normality and adjustment for multiple comparisons
☐	☒	A full description of the statistical parameters including central tendency (e.g. means) or other basic estimates (e.g. regression coefficient) AND variation (e.g. standard deviation) or associated estimates of uncertainty (e.g. confidence intervals)
☐	☒	For null hypothesis testing, the test statistic (e.g. F, t, r) with confidence intervals, effect sizes, degrees of freedom and P value noted<br>*Give P values as exact values whenever suitable.*
☒	☐	For Bayesian analysis, information on the choice of priors and Markov chain Monte Carlo settings
☒	☐	For hierarchical and complex designs, identification of the appropriate level for tests and full reporting of outcomes
☒	☐	Estimates of effect sizes (e.g. Cohen's d, Pearson's r), indicating how they were calculated

Our web collection on statistics for biologists contains articles on many of the points above.

Software and code

Policy information about availability of computer code

Data collection	Illumina HiSeq Control Software (2.7) was used to collect the high-throughput sequencing data in Genewiz (China)
Data analysis	High-throughputsequence analysis by Rgenome(http://www.rgenome.net/) and python, graphpad8.0;

For manuscripts utilizing custom algorithms or software that are central to the research but not yet described in published literature, software must be made available to editors and reviewers. We strongly encourage code deposition in a community repository (e.g. GitHub). See the Nature Portfolio guidelines for submitting code & software for further information.

Data

Policy information about availability of data

All manuscripts must include a data availability statement. This statement should provide the following information, where applicable:

- Accession codes, unique identifiers, or web links for publicly available datasets
- A description of any restrictions on data availability
- For clinical datasets or third party data, please ensure that the statement adheres to our policy

There is no restriction on the data associated with this study. High-throughput sequencing data have been deposited in the NCBI database (accession code PRJNA854910, PRJNA892719). Source data are provided with this paper.

Human research participants

Policy information about studies involving human research participants and Sex and Gender in Research.

Reporting on sex and gender	There were no human research participants in our research. So no sex or gender data were collected and analyzed.
Population characteristics	not mentioned
Recruitment	not mentioned
Ethics oversight	not mentioned

Note that full information on the approval of the study protocol must also be provided in the manuscript.

Field-specific reporting

Please select the one below that is the best fit for your research. If you are not sure, read the appropriate sections before making your selection.

- [x] Life sciences
- [ ] Behavioural & social sciences
- [ ] Ecological, evolutionary & environmental sciences

For a reference copy of the document with all sections, see nature.com/documents/nr-reporting-summary-flat.pdf

Life sciences study design

All studies must disclose on these points even when the disclosure is negative.

Sample size	No statistical methods were used to predetermine sample size for experiments. As described in Methods section, we added puromycin into culture medium for removing cells not transfected with the editing plasmids. The addition of antibiotics is mainly to ensure the tested cells containing the editing plasmids, and increase the uniformity of the editing efficiency data. To minimize other errors, we performed all prime editing experiments with the puromycin containing.
Data exclusions	No data was excluded from data analysis, and all data obtained was used in the statistic analysis.
Replication	Data was obtained in the fashion of biological replicates. All replicates performed in this study were biological replicates.
Randomization	For the transfected HEK293 cells,after puromycin screeing, all the remained live cell samples were collected for high-throughput sequencing. Therefore, the allocation was not relevant to this study.
Blinding	For the HEK293 cells, all the samples were collected for high-throughput sequencing. Therefore, the blinding was not relevant to this study.

Reporting for specific materials, systems and methods

We require information from authors about some types of materials, experimental systems and methods used in many studies. Here, indicate whether each material, system or method listed is relevant to your study. If you are not sure if a list item applies to your research, read the appropriate section before selecting a response.

Materials & experimental systems

n/a	Involved in the study	
x		Antibodies
	x	Eukaryotic cell lines
x		Palaeontology and archaeology
x		Animals and other organisms
x		Clinical data
x		Dual use research of concern

Methods

n/a	Involved in the study	
x		ChIP-seq
x		Flow cytometry
x		MRI-based neuroimaging

Eukaryotic cell lines

Policy information about cell lines and Sex and Gender in Research

Cell line source(s)	HEK 293T cells from ATCC
Authentication	No cell lines were authenticated
Mycoplasma contamination	Cells were not tested for mycoplasma contamination.
Commonly misidentified lines (See ICLAC register)	No cell lines were used that are in the ICLAC register.
